# Enhancing Benzo[a]pyrene Degradation by *Pantoea dispersa* MSC14 through Biostimulation with Sodium Gluconate: Insights into Mechanisms and Molecular Regulation

**DOI:** 10.3390/microorganisms12030592

**Published:** 2024-03-15

**Authors:** La Lai, Shuqi Li, Shaoping Zhang, Manchun Liu, Lianwei Xia, Yuan Ren, Tangbing Cui

**Affiliations:** 1 School of Biology and Biological Engineering, South China University of Technology, Guangzhou 510006, China; peng_na@foxmail.com (L.L.); 202121049986@mail.scut.edu.cn (S.L.); zzsspp2001@163.com (S.Z.); lmcmc@aliyun.com (M.L.); biscutsharlevi@mail.scut.edu.cn (L.X.); 2 School of Food and Pharmaccutical Engineering, Zhaoqing University, Zhaoqing 526000, China; 3 School of Environment and Energy, South China University of Technology, Guangzhou 510006, China; ceyren@scut.edu.cn

**Keywords:** biodegradation, Benzo[a]pyrene, sodium gluconate, *Pantoea dispersa* MSC14

## Abstract

We investigated biostimulation as an effective strategy for enhancing the degradation efficiency of recalcitrant organic compounds, with MSC14 (a novel polycyclic aromatic hydrocarbon degrading bacterium *Pantoea dispersa* MSC14) as the study material. Here, we investigated the impact of sodium gluconate on MSC14-mediated degradation of B[a]p. This study focused on the application of sodium gluconate, a biostimulant, on MSC14, targeting Benzo[a]pyrene (B[a]p) as the model pollutant. In this study, the novel PAHs-degrading bacterium *P. dispersa* MSC14 demonstrated the capability to degrade 24.41% of B[a]p after 4 days. The addition of the selected sodium gluconate stimulant at a concentration of 4 g/L stimulated MSC14 to degrade 54.85% of B[a]p after 16 h. Intermediate metabolites were analyzed using gas chromatography-mass spectrometry to infer the degradation pathway. The findings indicated that sodium gluconate promoted the intracellular transport of B[a]p by MSC14, along with the secretion of biosurfactants, enhancing emulsification and solubilization capabilities for improved B[a]p dissolution and degradation. Further analysis through transmission electron microscopy (TEM) and scanning electron microscopy (SEM) revealed the formation of a biofilm by MSC14 and an increase in flagella as a response to B[a]p stress. Transcriptome profiling elucidated the interplay of quorum sensing systems, chemotaxis systems, and flagellar systems in the degradation mechanism. Additionally, the study uncovered the molecular basis of B[a]p transport, degradation pathways, metabolic changes, and genetic regulation. In summary, the addition of sodium gluconate promotes the degradation of B[a]p by *P. dispersa* MSC14, offering the advantages of being rapid, efficient, and cost-effective. This research provides an economically viable approach for the remediation of petroleum hydrocarbon pollution, with broad potential applications.

## 1. Introduction

In recent years, various environments, including soil, oceans, and the atmosphere, have been frequently subjected to pollution by polycyclic aromatic hydrocarbons (PAHs) [1], which pose potential ecological and human health hazards due to their bioaccumulation and “three types of toxicity” (mutagenicity, teratogenicity, and carcinogenicity) [2]. Among the 16 priority PAHs regulated by the U.S. Environmental Protection Agency (U.S. EPA), B[a]p stands out as a heavy PAH with five aromatic rings, exhibiting high stability and resistance to biodegradation [3]. B[a]p is widely distributed in soil and water [4] and, as it is part of a group of carcinogens [2,3], it exerts its toxicity through bioaccumulation, affecting the physiological growth and development of both animals and humans. It induces oxidative stress and inflammation in the gastrointestinal tract, lungs, and liver [5,6,7]. Therefore, the elimination of B[a]p from the environment is an urgent issue.

The current remediation methods for B[a]p pollution mainly include chemical remediation, physical remediation, biological remediation, and the combined application of these three technologies [8]. However, traditional physical and chemical remediation technologies, such as soil washing, electrokinetics, and chemical oxidation [9], suffer from drawbacks such as technical complexity, high energy consumption, and secondary pollution. Biological remediation utilizes the metabolism of living organisms, especially microorganisms, to degrade pollutants in the environment into CO_2_ and water or convert them into non-toxic or less toxic substances that can re-enter the biogeochemical cycles of the Earth. Therefore, using microorganisms to degrade B[a]p is a more economically efficient method to address B[a]p pollution issues [10,11,12].

Currently, bacteria capable of degrading B[a]p that have been isolated mainly include *Pseudomonas* sp., *Bacillus subtilis*, and *Rhodococcus wratislaviensis*, among others. For instance, *R. wratislaviensis* achieves a B[a]p degradation rate of 28% after 7 days [13], *Pseudomonas* sp. Lphe-2 can degrade 53% of B[a]p after 10 days [14], and *Pseudomonas* sp. S5 achieves a B[a]p degradation rate of 44.02% after 15 days [15]. Additionally, the *actinomycete rjgii*-135 degrades 60% of B[a]p after 8 days [16]. Other bacterial strains have not been extensively studied due to factors such as their pathogenicity or lower degradation efficiency. Therefore, the current focus of research is on discovering more microorganisms capable of degrading B[a]p and enhancing their utilization and degradation efficiency.

One method to enhance the efficiency of B[a]p degradation is the addition of a biostimulant. Biostimulation refers to the addition of nutrients to enhance the activity of functional microorganisms [17,18]. The addition of biostimulants promotes rapid microbial growth, shortens remediation time, and increases degradation efficiency [19]. Studies have shown that improving the microbial bioavailability of hydrocarbons in the soil further enhances the microbial bioremediation capacity [20]. For example, Ali et al. [21] used alcohols and plant oils as biostimulants, increasing the B[a]p degradation rate from 36.9% to 54.7%. Li et al., by adding glucose as a co-substrate in the degradation system, achieved a 200% increase in the total petroleum hydrocarbon degradation rate in a microbial fuel cell [22]. Valentín et al. [23] found that the addition of 10–23 g/L of glucose resulted in *Bjerkandera* sp. BOS5, a white-rot fungus, achieving maximum degradation rates of 80.6%, 31.2%, 93.1%, and 83.4% for pyrene, anthracene, dibenzothiophene, and fluorene, respectively, within 18–26 days.

Further studies have demonstrated that biostimulants enhance the degradation efficiency by inducing the regulation of genes and enzymes associated with degradation [19]. Li et al. investigated the transcriptomic changes in *Pseudomonas aeruginosa* DN1 under anthracene stress, revealing genes involved in the metabolism of hydrocarbons and degradation of aromatic structures from heterologous biomass. The study highlighted differentially expressed genes in strain DN1’s anthracene degradation pathway, focusing on ABC transport systems, signal transduction, and gene expression regulation. Key enzymes in the degradation of phenanthrene, such as naphthalene dioxygenase gene (*nahAC*) and catechol 2,3-dioxygenase gene (*C23O*), were identified as critical in the process of phenanthrene degradation by *Pseudomonas fluorescens* AH-40 within 15 days [24]. Liu et al. [25] investigated the transcriptional changes of *Sphingomonas* GY2B during phenanthrene degradation under Tween-80 conditions. The study revealed that with increasing Tween-80 concentration, the expression of genes related to H^+^ transport increased, providing more energy for phenanthrene transmembrane transport. Additionally, genes involved in the phenanthrene degradation pathway and intracellular metabolic processes showed upregulation, enhancing the efficiency of GY2B in phenanthrene degradation.

Membrane transport is a crucial process for degradation, involving membrane proteins in transmembrane transport and signal transduction. Understanding the microscale mechanisms of PAHs degradation from the perspective of membrane proteins provides important insights into factors influencing the efficient degradation of PAH pollutants in soil and the theoretical and practical significance of using biotechnology for the remediation of PAH-polluted areas [26]. Jiang et al. [27] found that the addition of Tween-20 improved the degradation efficiency of anthracene by *Bacillus cereus*. These results suggested that Tween-20 could enhance bacterial motility while promoting the expression of certain transmembrane proteins, such as flagellar rod proteins and peptide ABC transport proteins.

Biostimulants, characterized by the use of inexpensive and common substances to stimulate microbial degradation of target pollutants, aim to achieve green, efficient, and non-secondary pollution goals. Studies have shown that the addition of anions of low molecular weight organic acids, such as succinate, oxalate, malonate, fumarate, and sodium gluconate could enhance the bioavailability of hydrocarbons by the soil microorganisms, and further enhance the bioremediation ability of microorganisms. Although various biostimulants have been used for the degradation of petroleum pollutants, current research primarily focuses on the dynamic changes in microbial communities, microbial activity, and the activity of specific degradation enzymes (such as peroxidases, lipases, and dehydrogenases) after adding biostimulants. However, there is a lack of comprehensive understanding of specific functional genes and their molecular mechanisms involved in the adsorption, transport, and degradation processes induced by biostimulants in the microbial petroleum degradation process.

Genomic and transcriptomic technologies are powerful tools for in-depth studies of microbial life activities and physiological processes. Genomics plays a crucial role in exploring degradation genes related to environmental pollutants and understanding microbial physiological processes, such as chemotaxis, transport, regulation, and signal transduction, involved in pollutant degradation processes. Recently, transcriptomic approaches have been used to study the mechanism of PAH degradation by bacteria. Transcriptomics can elucidate the differential gene expressions during physiological activities such as substrate transport, signal transduction, and metabolism under relevant physiological conditions, revealing genes involved in specific physiological processes and their transcriptional state changes [28].

Our laboratory previously isolated *P. dispersa* MSC14 using B[a]p as the sole carbon source from petroleum-contaminated soil in a mining area, demonstrating superior efficiency in B[a]p degradation compared to other microorganisms. In this study, using *P. dispersa* MSC14 as the subject and B[a]p as the target pollutant, we screened the biostimulant sodium gluconate, which significantly enhanced the efficiency of B[a]p degradation. We explored the molecular mechanisms by which sodium gluconate enhances the capability of MSC14 to degrade B[a]p. We not only examined its growth and degradation characteristics, but also analyzed the biological functions of differentially expressed genes (DEGs) in this strain using transcriptomics. Various methods were employed, including surfactant assays, gas chromatography-mass spectrometry for intermediate metabolite determination, and comprehensive experiments combining scanning electron microscopy and transmission electron microscopy to observe microbial morphological features. These findings provide new insights into the mechanism of sodium gluconate stimulation in the degradation of B[a]p by *P. dispersa* MSC14, offering a theoretical basis and governance strategy for enhancing the bioremediation of environmental pollutant B[a]p.

## 2. Materials and Methods

### 2.1. Chemicals

B[a]p (purity 97%) was purchased from the Leyan Chemical Company. Acetonitrile (purity ≥ 99.9%) was obtained from the Macklin Company. Sodium gluconate (purity > 98%) was obtained from the Macklin. Biological Technology Company (Guangzhou, China). The rest of the reagents were analytically pure unless otherwise specified.

### 2.2. Strain Screening

Soil samples from the Open-pit Mine Ecological Park (Maoming, China) were subjected to five rounds of dilution, shaken at 150 rpm and 30 °C for 12 h, followed by a 7-day enrichment culture. This process was repeated five times. The enriched bacterial liquid was then diluted at different concentrations and spread on plates for isolation. Multiple streaking was employed for purification, with individual colonies inoculated into LB liquid medium for overnight culture. The bacterial suspension was adjusted to OD600 = 1.0. Subsequently, a 10% volume of the seed solution was added to the screening medium, and the cultures were maintained for 6 days at 30 °C and 150 rpm in the dark. Strains exhibiting higher benzo[a]pyrene degradation rates were selected for sequencing.

### 2.3. B[a]p Biodegradation Experiments

*P. dispersa* MSC14 (GenBank accession number ON528714.1), belonging to the Gram-negative bacterium, was previously isolated from Open-pit Mine Ecological Park (Maoming, China). The strain was pre-cultivated in the mineral salt medium (MSM) with 20 mg/L B[a]p as the sole carbon source on a gyratory shaker (150 rpm) at 30 °C and used for further studies. During the stimulant screening experiment, seven biostimulants were added at concentrations of 1 g/L each, including salicylic acid, α-ketoglutaric acid, catechol, phthalic acid, gentisic acid, glucose, and sodium gluconate. The cultivation solution was spiked by firstly adding autoclaved MSM, and then adding an adequate volume of B[a]p stocked solution (1 g/L) dissolved in acetone to sterilized flasks. After evaporation of acetone, the strain was added. MSM consisted of the following components:(NH_4_)_2_SO_4_ 1 g/L, Na_2_HPO_4_·12H_2_O 2.017 g/L, KH_2_PO_4_ 0.2 g/L, MgSO_4_·7H_2_O 0.2 g/L, FeCl_3_·6H_2_O 6.25 mg/L, (NH_4_)_6_Mo_7_O_24_·4H_2_O 1 mg/L, CaCl_2_·2H_2_O 0.1 g/L.

### 2.4. B[a]p Detection

Residual B[a]p was extracted from MSM using liquid:liquid extraction (1:1 *v*/*v*, media: dichloromethane). The organic layer was separated and this process was repeated two times, filtered through anhydrous sodium sulfate, and pooled in the same flask. Samples were measured with high-performance liquid chromatography (HPLC). An Agilent TC-C18, 5 µm, 4.6 × 250 mm column was used, the mobile phase was 90% acetonitrile adjusted with a flow rate of 1 mL/min, and the UV detector was set at 290 nm wavelength. A B[a]p standard curve with R^2^ = 0.9996 was prepared, and the B[a]p concentration at different time points was measured according to the method described previously. To quantify the final extract, the curve between peak area and standard concentration was used. The biodegradation efficiency (BE) was determined by using Formula (1) [29]:

BE (%) = (Aac − As)/Aac × 100(1)

where Aac is the peak area of uncultured bacteria and As is the peak area of each sample.

### 2.5. Degradation Product Analysis

MSC14 bacteria were cultured in MSM for 0, 8, 16, 24, 32, 40, and 48 h. The culture medium was acidified by adding HCl, adjusting the pH to 2 using HCl with a concentration of 36.0% (*w*/*w*). Ethyl acetate was added in equal volumes, followed by shaking for 10 min, 30 kHz ultrasound for 10 min, triple extraction, and centrifugation for 5 min (5000 rpm). The resulting extract was added with an appropriate amount of Na_2_SO_4_ to remove moisture. The extract was evaporated to dryness, and 20 mL of methanol was added, followed by evaporation to 1 mL. Gas chromatography-mass spectrometry (Agilent Technologies, Santa Clara, CA, USA) was used for analysis with a capillary column (PH-5MS: 30 m × 0.25 mm × 0.25 µm). The temperature program involved initial temperature maintenance at 80 °C for 2 min, followed by an increase to 180 °C at a rate of 20 °C/min and maintenance at this temperature for 5 min. Subsequently, the temperature was increased to 300 °C at a rate of 10 °C/min and maintained for 20 min. Mass spectrometry conditions included electron impact (EI) ionization.

### 2.6. Effect of Sodium Gluconate on Bacterial Growth and Surface Characteristics

Scanning electron microscopy (SEM): Bacteria were incubated in MSM only, MSM+ B[a]p, MSM+ B[a]p +sodium gluconate for 8 h, respectively, then centrifuged and washed with phosphate buffer (0.1 M, pH 7.0). The samples were fixed with 2.5% glutaraldehyde for more than 4 h, washed three times in the phosphate buffer, then postfixed with 1% OsO4 in phosphate buffer for 1–2 h and washed three times in the phosphate buffer. The samples were dehydrated by a graded series of ethanol (30%, 50%, 70%, 80%, 90%, 95%, and 100%) for about 15 to 20 min at each step. Next, the samples were mixed with ethanol and isoamyl acetate (*v*/*v* = 1/1) for 30 min and then treated with pure isoamyl acetate for 1 h or left overnight. After critical point drying, the samples were coated with platinum and observed under a Hitachi SU8100 SEM.

Transmission electron microscope (TEM): After dehydration with ethanol, the samples were transferred to anhydrous acetone for 20 min as described earlier. They were placed in a 1:1 mixture of anhydrous acetone and final Spurr resin at room temperature for 1 h, then transferred to a 1:3 mixture of anhydrous acetone and final resin for 3 h, and transferred overnight to the final Spurr resin mixture. The specimens were placed in Eppendorf tubes containing Spurr resin and heated at 70 °C for more than 9 h. After sectioning, the sections were stained by uranyl acetate and alkaline lead citrate for 5 to 10 min, respectively, and observed in a Hitachi Model H-7650 TEM (Hitachi High-Tech, Shanghai, China).

### 2.7. Determination of Emulsifiability

The supernatant was collected after centrifuging 25 mL of the fermentation broth at 8000 rpm for 5 min. In total, 5 mL of liquid paraffin was added to the test tubes. After adding 5 mL of MSM and 5 mL of fermentation supernatant, respectively, the mixture was forcefully vortexed for 2 min. after rested for 1 h, 24 h, measuring the height of the emulsion layer (h1, cm) and the overall height of the liquid column (h, cm). Finally, the emulsion index was calculated with (2).

Emulsion Index (%) = h1/h × 100(2)

### 2.8. RNA-seq and RT-qPCR

The TRIzol method was utilized for bacterial RNA extraction, and the total RNA was split into two portions: one designated for RNA-seq and the other for RT-qPCR. The Nanodrop system and Agilent 2100 bioanalyzer determined RNA concentration and integrity, respectively. DNase I digested DNA in total RNA, followed by recovery using magnetic beads, and rRNA removal with the RNase H technique (Illumina, San Diego, CA, USA). The purified mRNA underwent fragmentation using a fragment buffer, and first-strand cDNA was generated through PCR. The second-strand cDNA was produced in a secondary reaction system, with product purification using magnetic beads. A-Tailing Mix and RNA Index Adapters were added for end repair, followed by PCR amplification and purification using Ampure XP Beads. Library quality was assessed with the Agilent Technologies (Santa Clara, CA, USA) 2100 bioanalyzer.

The splint oligo sequence denatured and circularized double-stranded PCR products, formed the final library as single-strand circular DNA. Phi29 (Thermo Fisher Scientific, Waltham, MA, USA) amplified the library, generating DNA nanoballs with over 300 copies per molecule. These nanoballs were loaded onto the patterned nanoarray, producing pair-end 100-base reads. SOAPnuke filtered sequencing data by removing reads with sequencing adapters, a low-quality base ratio (base quality ≤ 5) exceeding 20%, and reads with an unknown base ratio exceeding 5%. HISAT2 mapped clean reads to the reference genome, identifying fusion genes and differential splicing genes. RSEM calculated gene expression levels. For phenotype insight, Phyper performed GO and KEGG enrichment analyses on differentially expressed genes using the Hypergeometric test. DESeq2 conducted differential expression analysis with a Q-value threshold of ≤0.05. A rigorous Bonferroni-adjusted Q-value threshold of ≤0.05 corrected significant terms and pathways.

RNA for RT-qPCR underwent cDNA synthesis using the TransScript One-Step gDNA Removal and cDNA Synthesis SuperMix reagent kit. The primer sequences and gene names used in RT-qPCR are provided in Table S1, and the machine used was an Applied Biosystems StepOnePlusTM Real-Time System. The reaction system consisted of 20 μL, including 3 μL DNA template, 0.8 μL forward and reverse primers, 5.8 μL nuclease-free water, 10 μL 2 × transstart tip green qPCR supermix, and 0.4 μL passive reference dye (50×). The RT-qPCR reaction program consisted of an initial step at 95 °C for 30 s, followed by 45 cycles of 95 °C for 5 s and 60 °C for 34 s, After the cycling, the temperature was increased from 60 °C to 95 °C at a rate of 1.6 °C per second for 15 s. Then, the temperature was decreased from 95 °C to 60 °C for 60 s, Finally, the temperature was increased from 60 °C to 95 °C at a rate of 0.16 °C per second for 1 s. The expression levels of differentially expressed genes (DEGs) were normalized as using the expression levels of the gyrA gene, and the relative expression levels of DEGs were calculated using 2^−ΔΔCt^.

## 3. Results

### 3.1. Screening of Biostimulants

**Figure 1:**
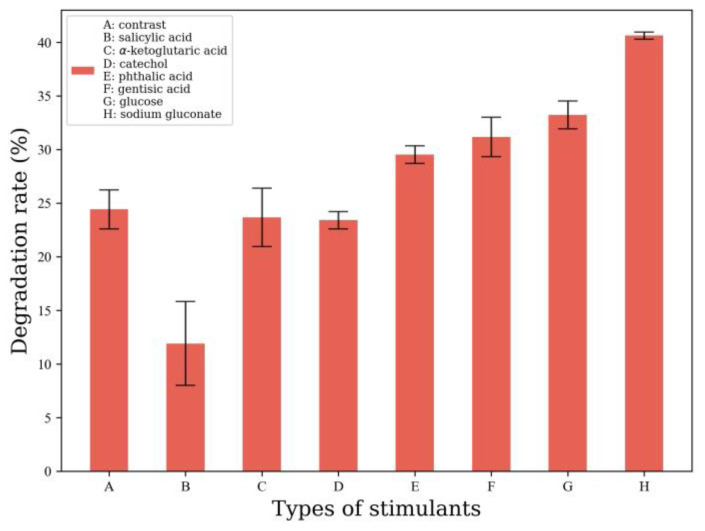
Screening of biostimulants.

To promote the degradation of B[a]p by MSC14, under 20 mg/L B[a]p stress and cultured for 4 days, 1 g/L, seven biostimulants, including salicylic acid, α-ketoglutaric acid, catechol, phthalic acid, gentisic acid, glucose, and sodium gluconate were added to the MSM medium of MSC14. The degradation rate of B[a]p was measured after 4 days, The degradation rate of MSC14 for benzopyrene was 24.41% without any biostimulant, and 44.2% with the addition of 1 g/L sodium gluconate (Figure 1). Therefore, we chose sodium gluconate as a biostimulant.

### 3.2. Sodium Gluconate Enhances Efficient Degradation of B[a]p by MSC14

**Figure 2:**
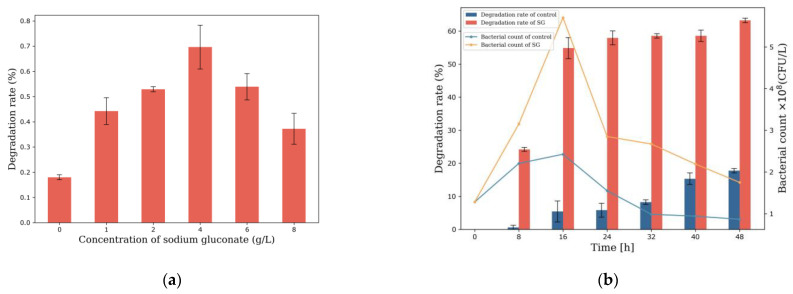
(**a**) The optimal concentration of sodium gluconate on enhancing the ability of MSC14 to degrade B[a]p: the red bars presents the degradation rate of MSC14 to 20 mg/L B[a]p at different sodium gluconate concentrations after 4 days; (**b**) the optimal culture time of sodium gluconate on enhancing the ability of MSC14 to degrade B[a]p.

To investigate optimal culture time of sodium gluconate on enhancing the ability of MSC14 to degrade B[a]p, sodium gluconate was added to MSC14 and cultured for 4 days. The results exhibited a concentration-dependent response to sodium gluconate. Optimal degradation was observed at a sodium gluconate concentration of 4 g/L, where MSC14 degraded 65.06% B[a]p after 4 days (Figure 2a). To investigate the influence of culture time and the ability of sodium gluconate enhancing MSC14 to degrade B[a]p, 4 g/L of sodium gluconate was added to MSC14 and cultured for different times (0, 8, 16, 24, 32, 40, 48 h), The bacterial count result showed that the group without the added sodium gluconate showed slow growth of MSC14 under B[a]p stress, with a significant decrease after 16 h, indicating evident toxicity of B[a]p to MSC14 (Figure 2b). In contrast, the group with added sodium gluconate demonstrated a substantial increase of MSC14 bacterial count, reaching its maximum value at 16 h. These results indicate that sodium gluconate could reduce the toxicity of B[a]p to MSC14. The bacterial count result showed that the degradation rate of B[a]p by MSC14 showed remarkable enhancement in degradation rate (913.2%) in 16 h under the influence of 4 g/L sodium gluconate, (Figure 2b). Therefore, 4 g/L of sodium gluconate stimulant was added to MSC14 and cultured for 16 h as transcriptome samples for follow-up study. The above results indicate that sodium gluconate effectively stimulates MSC14, enabling rapid degradation of B[a]p within a short time.

### 3.3. Gas Chromatography-Mass Spectrometry Analysis for Inferring Metabolic Pathways of Sodium Gluconate-Enhanced MSC14-Mediated B[a]p Degradation

**Figure 3:**
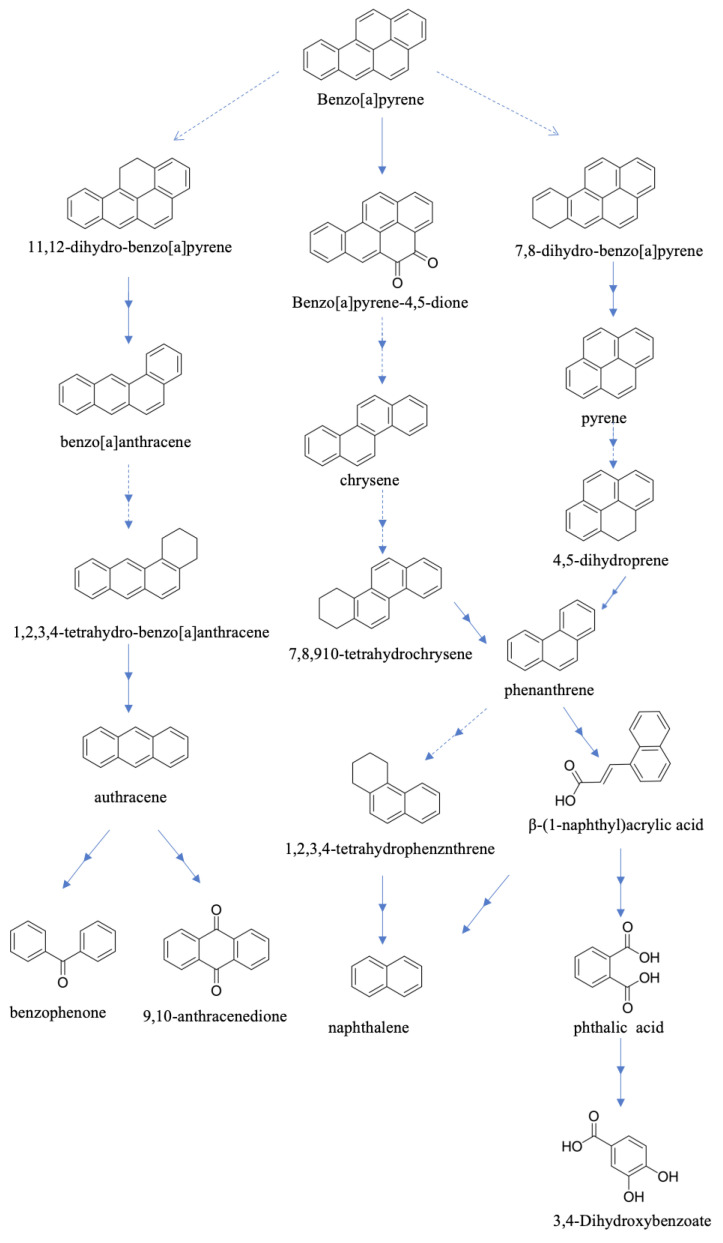
Metabolic pathways of B[a]p degradation by MSC14 as inferred from intermediate metabolites.

The metabolites in the metabolic process were further analyzed by GC-MS, and the results of B[a]p were compared with the chromatographic retention time of the actual compounds in the mass spectrometry as well as the retention time of molecular and fragment ions. Various intermediate substances such as B[a]p, anthracene, pyrene, phenanthrene, and derivatives of phthalic acid (Figures S1 and S2), and a significant amount of methylated intermediates were observed in this experiment, indicating that, in the presence of sodium gluconate, the strain *P. dispersa* MSC14 initiates the degradation of B[a]p through methylation activation reactions. The identified products allowed the inference of the metabolic pathway of B[a]p degradation by MSC14 (Figure 3) [30]. Under sodium gluconate stimulation, the degradation pathway of B[a]p by *P. dispersa* MSC14, MSC14 involved enzymatic activity on carbon atoms 7 and 8 of B[a]p. This activates a reaction leading to phenanthrene, which is subsequently oxidized to phenanthrenequinone. The surrounding benzene ring is oxidized to form naphthalene derivatives. This derivative undergoes decarboxylation and hydroxylation reactions to generate derivatives of phthalic acid. Finally, the product 3,4-dihydroxybenzoic acid ester is isomerized to succinyl coenzyme A and enters the tricarboxylic acid (TCA) cycle. Additionally, the second pathway detected the presence of B[a]p-4,5-dione, where enzymes act on carbon atoms 4 and 5 of B[a]p, leading to the formation of phenanthrene, and subsequent reactions are consistent with the first pathway. The third pathway involves enzymes acting on carbon atoms 11 and 12 of B[a]p, resulting in the formation of benzo[a]anthracene, which is oxidized to anthracene. Based on the corresponding detected products, the benzene ring oxidation in anthracene can produce one or two carbonyl groups.

### 3.4. Morphological Analysis of P. dispersa MSC14

**Figure 4:**
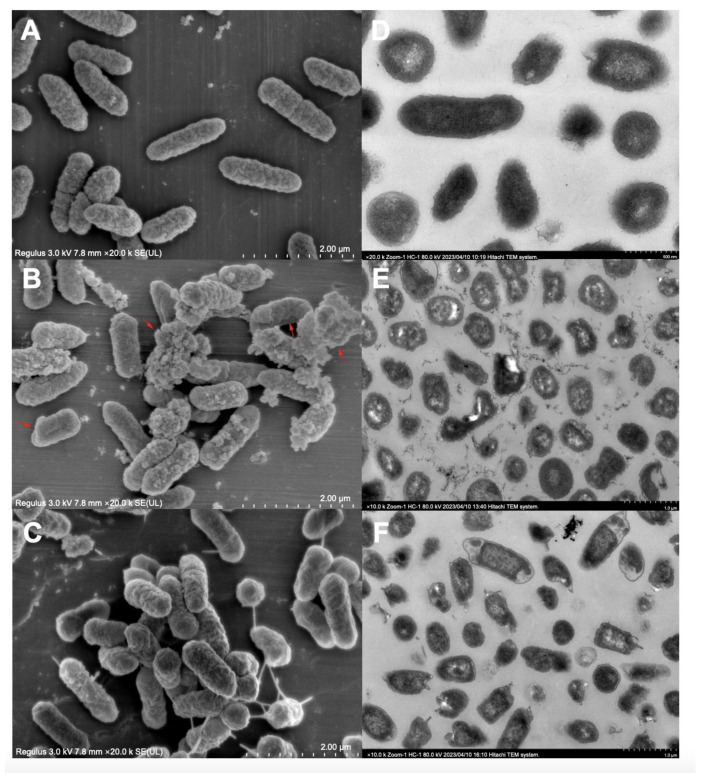
TEM (**Left**) and SEM (**Right**) scan for bacterial morphology. (**A**,**D**): blank group (the natural growth state of MSC14 without B[a]p stress); (**B**,**E**): control group (growth state of MSC14 under B[a]p stress); (**C**,**F**): Experimental group (growth state of MSC14 under B[a]p stress after adding sodium gluconate).

Microbial morphological changes are significant indicators of their adaptation to environmental stress. TEM observations of MSC14 strains under different treatments revealed distinct morphological alterations. When cultivated in pure inorganic salt me-dium, MSC14 bacteria exhibited a rod-shaped morphology, and a relatively dispersed distribution (Figure 4A). Under B[a]p stress, Extracellular polymer fragments appeared on the thallus surface (Figure 4B,E), which we considered was B[a]p adsorbed on the thallus surface. After the addition of sodium gluconate, bacteria gathered and the number of flagella increased. The number of extracellular polymers was greatly reduced, combined with the upregulation of the ABC transport system gene. We speculated that sodium gluconate could promote the adsorption and transport of B[a]p by MSC14 (Figure 4C,F) [31].

### 3.5. Emulsification and Hydrophobicity Analysis

**Figure 5:**
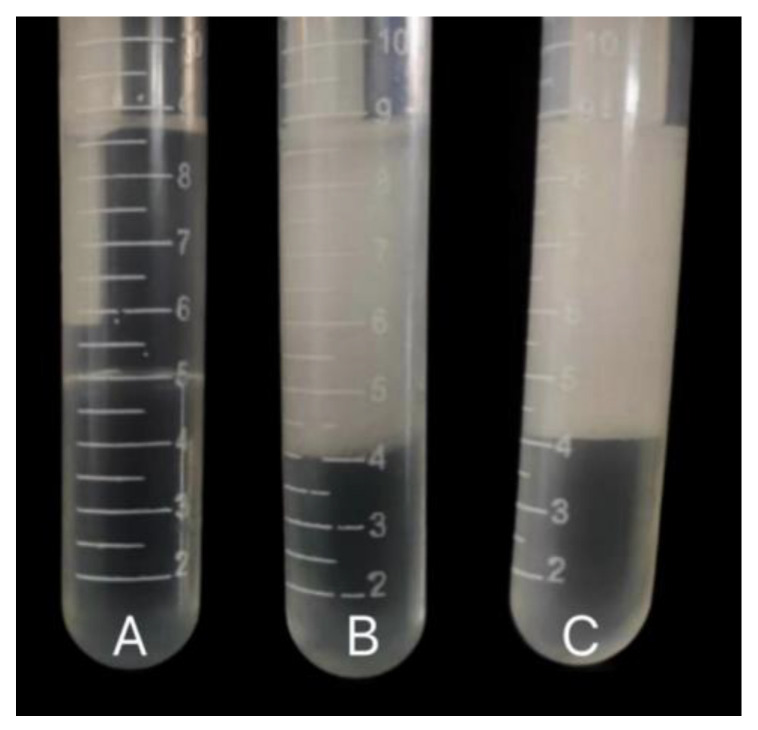
Analysis of surfactants in MSC14 products. (**A**) MSM+B[a]p; (**B**) MSM+ sodium gluconate; (**C**) MSM+B[a]p+sodium gluconate.

The analysis of emulsification and hydrophobicity in the culture medium revealed distinct observations. Under control conditions after 8 h of cultivation, no evident emulsified layer was observed in the culture medium. Conversely, under the presence of sodium gluconate, a pronounced emulsified layer became apparent after 8 h of cultivation (Figure 5). The emulsification index (recorded as EI-1 and EI-2) was determined at 1 h and 24 h, respectively. The biological emulsifiability (ES) was calculated, and the result was greater than 50% (Table 1). This indicates that sodium gluconate has the ability to stimulate MSC14 to produce biosurfactants and the emulsion was stable. Biosurfactants, acting as emulsifiers, can alter cell membrane permeability, enhance the migration ability of PAHs, and consequently improve the efficiency of degradation, making them an indispensable technique in bioremediation processes. Previous studies, such as that by Zang et al. demonstrated increased bioavailability and enhanced PAH degradation efficiency of *P. aeruginosa* when biosurfactant-producing strains were added to coking wastewater and sludge [32]. This suggests significant potential and practical value of biosurfactants in remediating environments contaminated with PAHs.

### 3.6. Transcriptome Analysis

#### 3.6.1. Analysis of Differentially Expressed Genes in MSC14 Stimulated by Sodium Gluconate for B[a]p Degradation

**Figure 6:**
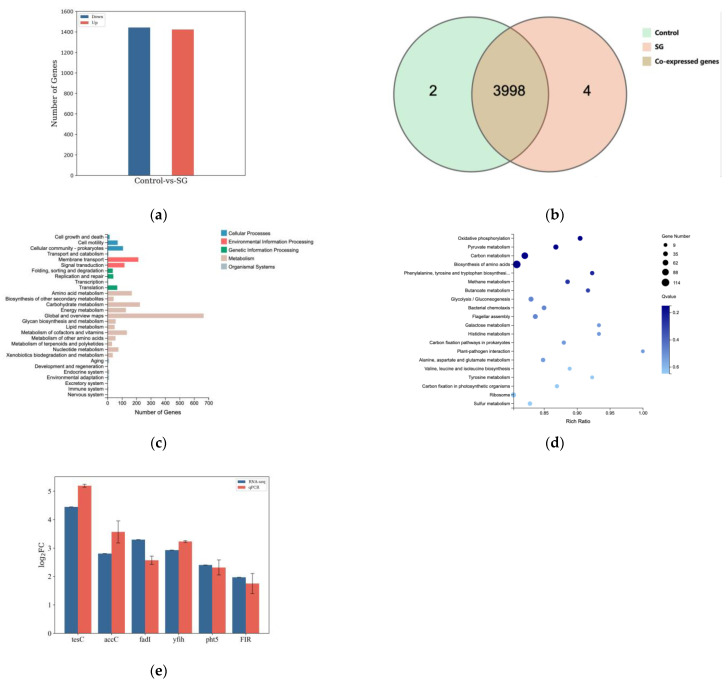
Transcriptional basic characteristics. Control represents the control group, SG represents the experimental group supplemented with sodium gluconate. (**a**) Statistics of the number of DEGs; (**b**) Venn diagram of inter-group expression levels: Analysis illustrates the overlap in gene expression between the experimental group treated with sodium gluconate and the control group during the degradation of B[a]p by MSC14. The brown area represents a total of 3998 genes expressed in common between the two groups. The experimental group (orange) and the control group (green) each have 4 and 2 uniquely expressed genes, respectively; (**c**) KEGG annotation classification of DEGs; (**d**) KEGG pathway enrichment analysis of DEGs; (**e**) RNA-seq and RT-qPCR. Figure 6 was created with biorender.com.

Utilizing DEseq2 (*p* < 0.05 and |log_2_FoldChange| > 0.0), gene differential expression analysis revealed that under B[a]p stress conditions, 2865 genes exhibited significant differences in expression levels between the control group (without the addition of sodium gluconate) and the experimental group (with sodium gluconate added) (Figure 6a). Among these, 1423 genes were upregulated, and 1442 genes were downregulated. Further Venn diagram analysis showed that 3998 genes were commonly expressed between the two groups, with 4 genes uniquely expressed in the experimental group (Figure 6b). To gain a deeper understanding of the functions of differentially expressed genes (DEGs), KEGG annotation and enrichment analysis were performed (Figure 6c,d). The DEGs were mainly enriched in pathways related to amino acid synthesis, carbon metabolism, oxidative phosphorylation, quorum sensing systems, chemotaxis systems, two-component systems, ABC transporters, and other processes. To verify the accuracy of differential gene expression levels in the RNA-seq results, seven differentially expressed genes in RNA-seq were selected for RT-qPCR validation (Figure 6e). These results were consistent with the RNA-seq, demonstrating the reliability of RNA-seq analysis. Firstly, the ABC transport system gene was significantly upregulated (Table S2), combined with the results of 3.3, it indicated that sodium gluconate can stimulate the synthetic layer of the ABC transporter protein, and B[a]p was more transported into the cell through the ABC transport system than other transporters. Secondly, key genes involved in various metabolic pathways associated with B[a]p degradation showed significant upregulation, including *PIR, PHT5, fadA, fadB, fadI, fadJ, yfiH,* etc. (Table 2). These results indicate that sodium gluconate promotes MSC14’s degradation of B[a]p through multiple metabolic pathways.

#### 3.6.2. Changes in Metabolic Pathways Stimulated by Sodium Gluconate in MSC14

**Figure 7:**
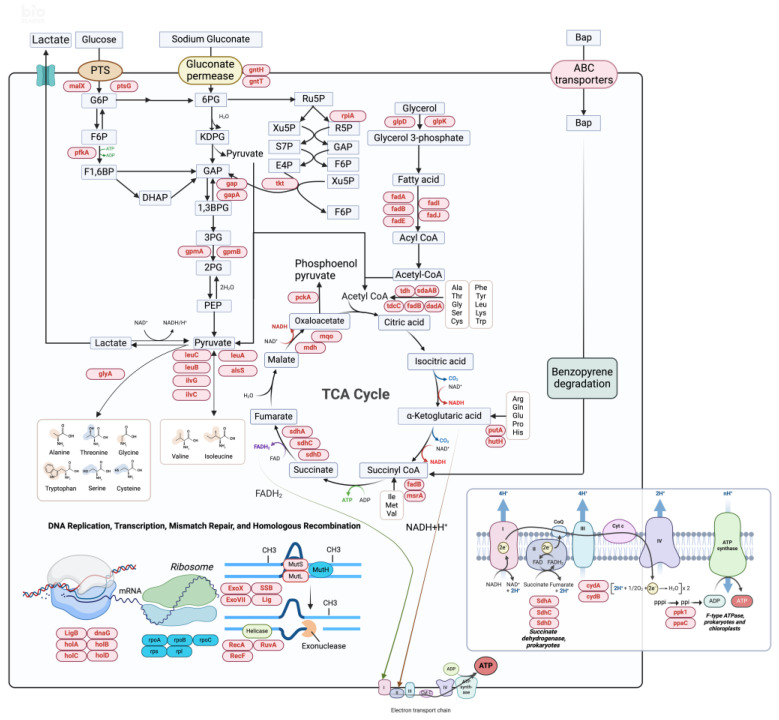
DEGs analysis of MSC14 stimulated by sodium gluconate. The genes highlighted within the red boxes in the figure indicate upregulated genes, while those within the blue boxes represent downregulated genes, the blue box represents substances related to metabolism, The brown box represents amino acids, Enlarged illustration of the Electron transport chain in the lower-right quadrant of the image. Figure 7 was created with biorender.com.

The degradation of B[a]p in MSC14 involves numerous genes and, compared to the control group, many genes exhibit significant differential expression. An overview diagram of the expression of these genes is shown in Figure 7, with detailed information on metabolism described below.

##### Entner–Doudoroff (ED) Pathway

The Entner–Doudoroff pathway, also known as the 2-keto-3-deoxy-6-phosphogluconate (KDPG) pathway, is a glucose-specific induced pathway responsible for the rapid breakdown of glucose salts, facilitating glucose salt transport and metabolism [33]. When sodium gluconate is used as the carbon source for MSC14, the expression levels of relevant genes involved in the KDPG pathway are significantly upregulated (Table S3). This includes two genes, *GntI* and *GntII*, associated with the *Gnt* system. *GntI* (Gnt-I system high-affinity gluconate transporter) that encode a high-affinity glucose salt transporter, ensuring efficient transport even at lower glucose salt concentrations [34]. It recognizes and binds sodium gluconate, transporting it from the environment into the cell. *GntII* transcriptionally regulates the *Gnt* system, and the simultaneous high expression of both genes promotes the transport of sodium gluconate and its phosphorylation. This process involves the conversion of sodium gluconate into gluconic acid-6-phosphate, and 2-keto-3-deoxy-6-phosphogluconate is cleaved and reduced to 3-phosphoglyceraldehyde and pyruvate by KDPG aldolase. Subsequently, 3-phosphoglyceraldehyde can enter the EMP pathway and be converted to pyruvate, entering subsequent metabolic pathways.

##### PTS and Central Carbon Metabolism Pathways

The phosphotransferase system (PTS) is a widely present intracellular substance transport pathway in *Bacteria* and *Archaea*, and is responsible for transporting specific carbon sources into the cell for irreversible phosphorylation [35]. Upon the addition of sodium gluconate, the expression levels of relevant genes in the PTS pathway within MSC14 are significantly upregulated (Table S3). This includes *ptsG* (encoding acid phosphatase for nucleoside transport) and *malX* (encoding lead enzyme to facilitate microbial substrate transport into the cell) [36]. The upregulation of these genes promotes the effective absorption, conversion, and metabolism of sugar molecules (nucleosides, fructose, etc.).

Furthermore, when sodium gluconate serves as the sole carbon source for MSC14, the transcription levels of most genes in glycolysis and the tricarboxylic acid cycle (TCA cycle) are also significantly upregulated (Table S4). This includes *pfkA* (encoding 6-phosphofructokinase), *gap* and *gapA* (catalyzing glyceroldehyde-3-phosphate), *fba* (catalyzing fructose-1,6-diphosphate conversion to glyceroldehyde-3-phosphate) and *gpmA* and *gpmB* (catalyzing 2,3-diphosphoglycerate conversion to 3-phosphoglycerate). The upregulation of these genes accelerates glycolytic metabolism and subsequently speeds up downstream TCA cycle pathways (Figure 7). In the TCA cycle, the expression levels of genes in the succinate dehydrogenase (*SDH*) family are significantly upregulated, producing ample ATP through oxidative phosphorylation to provide metabolic energy [37]. Additionally, the upregulation of *mqo* and *mdh* (catalyzing malate and oxaloacetate metabolism) regulates the balance between malate and oxaloacetate, driving the progression of the TCA cycle. Therefore, sodium gluconate enhances the central carbon metabolism of glycolysis and the TCA cycle in MSC14, enabling adaptation to the stress of B[a]p.

##### Glycerol Metabolism

Glycerol metabolism is one of the main ways of energy metabolism, and *glpD* (glycerol-3-phosphate dehydrogenase) and *glpK* (glycerol kinase, the rate-limiting enzyme of glycerol metabolism) are critical genes for glycerol metabolism (Figure 7). Under the stimulation of the biostimulant sodium gluconate, the expression levels of *glpD* and *glpK* genes in MSC14 are upregulated (Table S5), meeting the high energy demand for B[a]p degradation.

##### Amino Acid Metabolism Pathways

Under sodium gluconate stimulation, the amino acid metabolism genes level increases in the MSC14 strain (Table S6), such as *hutH* (histidine degradation), *hutC* (GntR family transcriptional regulator), *hutG* (N-formylglutamate deformylase), and *hutI* (imidazolonepropionase), promoting the utilization and metabolism of amino acids, maintaining cellular homeostasis, and providing energy to cope with nutrient deficiencies in the environment.

##### Fatty Acid Metabolism

Fatty acids, through beta-oxidation, can generate abundant acetyl-CoA and ATP. The genes encoding key proteins in this process, such as *fadA* (encoding acetyl-CoA acetyltransferase), *fadB* (responsible for beta-oxidation in fatty acid metabolism) [38], and *fadN* (fatty acyl-CoA oxidation), all show increased expression levels in sodium gluconate-treated MSC14 (Table S6) (Figure 7). This suggests that the addition of sodium gluconate enhances the fatty acid metabolism of MSC14, producing more energy to adapt to the stress environment of B[a]p.

#### 3.6.3. Sodium Gluconate Promotes Oxidative Phosphorylation for ATP Production in MSC14

Oxidative phosphorylation is the primary metabolic pathway for ATP generation, Through DEG analysis, it is observed that under sodium gluconate treatment, the expression levels of *sdh* (involved in the electron transport chain) [39], *cyd* (involved in oxygen reduction) [40] and *ppk* (involved in polyphosphate synthesis) [41] genes in MSC14 are all upregulated (Table S7). This indicates that sodium gluconate stimulation enhances the oxidative phosphorylation system in MSC14, producing more energy to sustain normal life activities, which is crucial for the degradation of B[a]p.

#### 3.6.4. Sodium Gluconate Stimulates DNA Replication Upregulation and Transcription Downregulation in MSC14

DNA replication and transcription are fundamental biological processes in living organisms, regulating cell growth and replication. In this study, under sodium gluconate stimulation, almost all genes encoding DNA replicase in B[a]p-stressed MSC14 were significantly upregulated (Table S7), including *ligB* (DNA ligase), *holABCD* [42,43] (DNA polymerase III accessory proteins), and *dnaG* (responsible for RNA primer synthesis on both template strands at replication forks during chromosomal DNA synthesis). The expression levels of *rpo* series enzyme genes encoding RNA polymerase and *rps* and *rpl* related to ribosomes were significantly downregulated (Table S8). For DNA transcription, studies have shown that when bacteria are exposed to adverse or harmful environmental conditions, transcription levels are downregulated to adapt to different environments. These results indicated that sodium gluconate promoted DNA replication of the MSC14 strain under B[a]p stress and reduced unnecessary synthesis in order to adapt to the stressed environment.

#### 3.6.5. Sodium Gluconate Stimulates Homologous Recombination and DNA Repair in MSC14

In prokaryotes, DNA mismatch repair (MMR) significantly contributes to genome integrity by rectifying mismatched bases primarily arising from replication errors. Homologous recombination is a crucial mechanism for DNA repair and genetic recombination. Under the stimulation of sodium gluconate, the expression levels of genes encoding repair and recombination enzymes in the MSC14 strain subjected to B[a]p stress were upregulated (Table S8). These include *mutS* (searches for and recognizes mismatches and lesions in DNA), *mutL* (a widely conserved nicking endonuclease [44]), *ligB* (catalyzes the formation of phosphodiester bonds) [45], *xseB* (a single-strand-specific deoxyriboexonuclease), *SSB* (selectively binds to ssDNA with high affinity and little sequence specificity, protecting ssDNA from degradation and recruiting other proteins necessary for DNA replication, recombination, and repair [46]), *recF* (participates in DNA chain recombination), *ruvA* [47] (responsible for DNA branch migration), and *recA* (mediates DNA damage repair and genetic recombination). (Figure 7) The upregulation of these genes indicates that glucose sodium gluconate promotes DNA damage repair in the MSC14 strain under B[a]p stress in the environment.

#### 3.6.6. Regulation of Quorum Sensing Systems in MSC14 Stimulated by Sodium Gluconate

**Figure 8:**
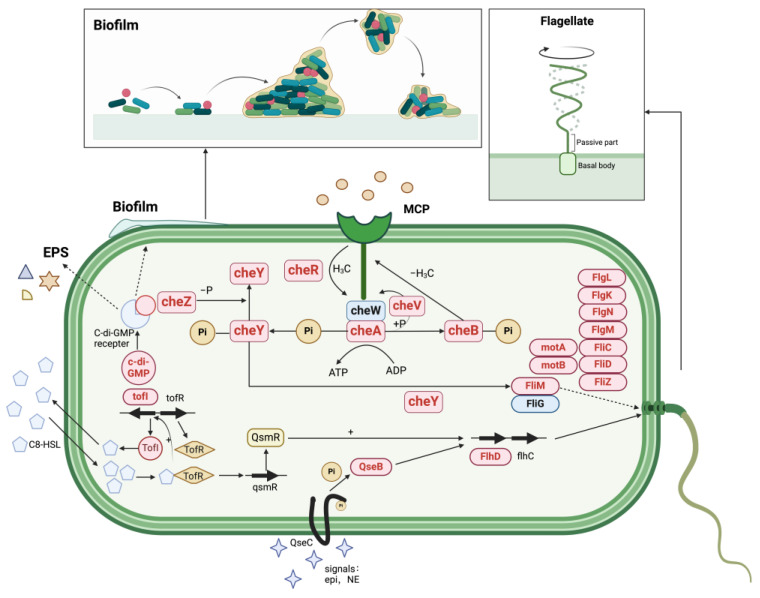
Regulation of MSC14 QS system stimulated by sodium gluconate. The genes highlighted within the red boxes in the figure indicate upregulated genes, while those within the blue boxes represent downregulated genes.

Sodium gluconate and B[a]p initiate signal transduction as the first extracellular messenger. The carrier protein enters the cell and induces the production of the second messenger molecule, thus acting on the intracellular target protein, regulating gene expression or enhancing functional enzymes. In addition, membrane protein sensors detect the extracellular first messenger and phosphorylate intracellular proteins, thereby regulating gene expression [48]. Microbial quorum sensing (QS) refers to the continuous production of small molecules known as autoinducers (AI) by bacteria during their growth. These AIs are secreted into the environment and increase with the growth of bacterial population. When the concentration of AIs in the environment reaches a certain threshold, they enter the bacterial cells and bind to specific receptors, thereby regulating intracellular signaling pathways. Current research indicates that QS plays a crucial role in promoting organisms’ adaptation to complex environments, enhancing the cycling, bioutilization, detoxification, absorption, transport, and transformation of organic pollutants [49,50]. Various microbial behaviors, such as bioluminescence, secretion of extracellular polysaccharides, biofilm formation, and bacterial migration, have been confirmed to be regulated by QS phenomena [51,52]. Based on the results of this study, genes associated with the QS system are upregulated during degradation (Table S9). Regulating microbial physiological characteristics, we propose the regulatory mechanism of the quorum sensing system in MSC14 stimulated by sodium gluconate under B[a]p stress (Figure 8). Detailed information is described below.

##### Chemotaxis System and Flagellar Movement

Bacterial chemotaxis refers to the directed movement of bacteria towards beneficial chemical gradients or away from toxic chemical gradients. This movement is primarily achieved through the rotation of flagella, a behavior with significant implications in signal transduction, biofilm formation, and pollutant degradation. Flagellar rotation and chemotaxis involve multiple genes (Table S10), including the MS ring (*FliF*), cytoplasmic output components (known as the C ring–*FliG*, *FliM*, and *FliN*), periplasmic space-spanning rod (*FlgB*, *FlgC*, *FlgF*, and *FlgG*), peptidoglycan layer-related P ring (*FlgI*), outer membrane-associated L ring (*FlgH*), and motor proteins (*MotA* and *MotB*) [26,28].

Under sodium gluconate stimulation, genes related to flagella and chemotaxis systems in MSC14 were upregulated under B[a]p stress (Table S10). In the two-component system, *CheA*, through autophosphorylation, transfers phosphorylation to the regulatory protein *CheY*, which controls bacterial flagellar rotation. Phosphorylation of *CheY*’s *Asp57* leads to reduced *CheA* affinity and increased interaction with the *FliM* protein, enhancing flagellar clockwise rotation. Therefore, we hypothesize that sodium gluconate promotes the expression of flagellar rotation genes in MSC14 under B[a]p stress, increasing the number and rotation of flagella to cope with the stressful B[a]p environment.

##### Two-Component System

The two-component system is a signal transduction system present in bacteria, enabling them to sense changes in the external environment and regulate survival and expression of virulence factors. The key regulatory proteins in the two-component system include histidine protein kinase (*HPK*, located in the cytoplasmic membrane, responsible for sensing and transmitting signals) and *OmpR* (response regulator, regulating the expression of outer membrane proteins). Upon receiving signals, *HPK* phosphorylates and transfers signals to downstream *OmpR*, which, when phosphorylated (*OmpR*~P), transcriptionally regulates outer membrane proteins to enhance the permeability of the outer membrane in response to environmental stress [53]. This study also found that under sodium gluconate stimulation, the expression of genes encoding *HPK* and *OmpR* proteins in MSC14 under B[a]p stress were upregulated (Table S11), indicating that sodium gluconate increases the membrane permeability of MSC14, accelerating the absorption and degradation of B[a]p by MSC14.

##### Sodium Gluconate Stimulates the Production of Biosurfactants in MSC14

Biological surfactants are metabolites secreted by microorganisms during certain metabolic processes that exhibit surface activity. They can promote the dissolution of B[a]p by emulsification or solubilization [54], making it more accessible for microbial degradation. Experiments show that sodium gluconate can stimulate MSC14 to produce biosurfactants (Figure 4). Sodium gluconate can regulate fatty acid synthesis, and genes related to fatty acid anabolic metabolism, *tesC, fadL, fadI, fadJ, fadR, fadD, fadA, fadB, TesC, FadL, FadI,* and *FadJ*. The upregulation of adeE can promote the synthesis of biosurfactants, thereby emulsifying B[a]p and improving the degradation efficiency; the upregulation of *OmpA* with high emulsification can promote the bioavailability of hydrophobic hydrocarbons and thus increase the degradation efficiency of B[a]p [55]. The functions of cell movement, EPS secretion, and biofilm formation regulated by *c-di-GMP* (cyclic diguanosine) are also controlled by the quorum sensing system. Foreign research groups have found that the HapR protein in the quorum sensing system of Vibrio cholerae can inhibit the expression of c-di-GMP synthesis genes, and indirectly inhibit the formation of biofilm by promoting the degradation of c-di-GMP [56]. This regulatory mechanism plays a role in the modulation of gene expression, metabolic enzyme activity, and the formation of biofilms. In addition, rhl in QS systems can regulate the production of the biosurfactant rhamnoolipid. The adsorption and degradation effects of rhamnoolipid on phenanthrene-degrading bacteria *Pseudomonas* sp. Ph6 have been reported [57]. Both experimental results and transcriptome results showed that sodium gluconate promoted the formation of biosurfactant in MSC14, enhanced the emulsification of PAHs, increased its bioavailability, and directly promoted the degradation of B[a]p.

## 4. Discussion

This study selected the biostimulant sodium gluconate and investigated its effects and mechanisms in promoting the degradation of B[a]p by the newly discovered B[a]p-degrading bacterium *P. dispersa* MSC14 in our laboratory for the first time. The following conclusions were drawn:

(1) Currently, bacteria capable of degrading B[a]p that have been isolated mainly include *Pseudomonas*.sp, *B. subtilis*, and *R. wratislaviensis*, among others. For instance, *R. wratislaviensis* achieves a B[a]p degradation rate of 28% after 7 days [13], *Pseudomonas* sp. Lphe-2 can degrade 53% of B[a]p after 10 days [14], and *Pseudomonas* sp. S5 achieves a B[a]p degradation rate of 44.02% after 15 days [15]. Additionally, the *Actinomycete* rjgii-135 degrades 60% of B[a]p after 8 days [16]. In this study, the novel PAHs-degrading bacterium *P. dispersa* MSC14 demonstrated the capability to degrade 24.41% of 20 mg/L B[a]p after 4 days. The addition of the selected sodium gluconate stimulant at a concentration of 4 g/L stimulated MSC14 to degrade 54.85% of B[a]p after 16 h, the level of B[a]p decreased during from 20 mg/L to 10.97 mg/L. This efficiency surpasses that of many other PAHs-degrading bacteria, exhibiting a shorter degradation time and higher effectiveness.
(2) Metabolites were detected through GC-MS to infer the degradation pathway of B[a]p by the strain *P. dispersa* MSC14 under sodium gluconate stimulation. Concurrently, transcriptome analysis revealed a significant upregulation of enzymes related to B[a]p degradation, such as *yfiH, pht5,* and *PIR*, collectively promoting the degradation of B[a]p. This elucidates the process by which MSC14 degrades B[a]p into salicylic acid salts, entering the TCA cycle, and providing crucial insights into the mechanism of B[a]p degradation by MSC14.
(3) TEM and SEM experiments observed bacterial morphological changes in MSC14 under B[a]p stress. After the addition of sodium gluconate, bacteria gathered, the number of flagella increased, and the number of extracellular polymers was greatly reduced. Combined with the upregulation of the ABC transport system gene, we speculate that sodium gluconate could promote the adsorption and transport of B[a]p by MSC14.
(4) Transcriptome-level analysis elucidated the regulation of metabolic pathways in MSC14 during B[a]p degradation under sodium gluconate stimulation. This included pathways such as the Entner–Doudoroff (ED) Pathway, central carbon metabolism pathways, oxidative phosphorylation, and also involved gene changes related to DNA repair, recombination, replication, and transcription. This study further explained, at the molecular level, how sodium gluconate stimulation modulates MSC14’s metabolic levels, substance transport, energy metabolism, and genetic material to enhance its ability to adapt to B[a]p stress.
(5) DEGs results indicated a significant upregulation of genes related to the quorum sensing (QS) system in MSC14 during B[a]p degradation under sodium gluconate stimulation. QS plays a crucial role in promoting microbial adaptation to complex environments and the circulation, bidirectional utilization, detoxification, absorption, transport, and transformation of organic pollutants. This study proposed the regulatory mechanism of the QS system in MSC14 under sodium gluconate stimulation during B[a]p stress, further detailing the interplay of extracellular polysaccharide secretion, biofilm formation, surfactant production, and flagellar movement in the QS system, providing new insights for enhancing B[a]p degradation.
(6) Surfactant experiments and transcriptome analysis revealed that under B[a]p stress, sodium gluconate could promote MSC14 to secrete biosurfactants. Previous research has indicated a direct correlation between biosurfactants and the degradation of polycyclic aromatic hydrocarbons [32]. Surfactants enhance emulsification and solubilization, thereby promoting the dissolution and degradation efficiency of B[a]p. Hence, this is considered one of the crucial mechanisms by which sodium gluconate stimulates MSC14 to degrade B[a]p.

In this study, we significantly increased the degradation efficiency of MSC14 towards B[a]p by using sodium gluconate as a biostimulant. Additionally, to investigate the relevant mechanisms by which sodium gluconate, as a biostimulant, enhances the degradation efficiency of B[a]p, we not only examined its growth and degradation characteristics but also analyzed the biological functions of differentially expressed genes (DEGs) in this strain using transcriptomics. Furthermore, we employed various methods, including surfactant experiments, gas chromatography-mass spectrometry for determining intermediate metabolites, and SEM and TEM for observing the morphological characteristics of microorganisms. This comprehensive approach provides new insights into the mechanism of sodium gluconate-stimulated B[a]p degradation by the novel polycyclic aromatic hydrocarbon-degrading bacterium *P. dispersa* MSC14. Subsequently, we will conduct field experiments to explore the practical application effects of sodium gluconate as a stimulant in promoting MSC14 to degrade B[a]p, offering specific strategies for B[a]p degradation.

## Figures and Tables

**Table 1:** Determination of emulsifiability of MSC14.

	EI-1 h	EI-24 h	ES = (EI − 24/EI − 1) × 100
A. MSM+B[a]p	0	0	0
B. MSM+Sodium gluconate	0.52	0.50	96%
C. MSM+B[a]p+Sodium gluconate	0.53	0.51	96%

**Table 2:** DEGs involved in B[a]p degradation.

Gene ID	Symbol	Description	Log2FC
K7H94_RS13370	*PHT5*	4,5-dihydroxyphthalate decarboxylase, Phenanthocyanin metabolism catalyzes 4,5-dihydroxyphthalates to undergo hydroxyl cleavage to produce protocatechuic acid	2.40
K7H94_RS02335	*yfiH*	aldehyde dehydrogenase, Oxidize phenolic compounds to quinones	2.92
K7H94_RS09205	*PIR*	quercetin 2,3-dioxygenase, Catalyze the oxidation of quercetin 2,3	1.96
K7H94_RS17420	*fadA*	Acetyl-CoA acyltransferase	1.35
K7H94_RS17415	*fadB*	Enoyl-CoA isomerase	2.27
K7H94_RS05995	*fadI*	Acetyl-CoA acyltransferase	3.29
K7H94_RS06000	*fadJ*	Enoyl-CoA isomerase	1.25
K7H94_RS02435		SDR family oxidoreductase	2.83

## Data Availability

Data are contained within the article and Supplementary Materials.

## Supplementary Materials

The following supporting information can be downloaded at: https://www.mdpi.com/article/10.3390/microorganisms12030592/s1, Figure S1: The intermediate metabolites of B[a]p degraded by MSC14 measured by GC-MS; Figure S2: The intermediate metabolites of B[a]p degraded by MSC14 measured by GC-MS; Table S1: Primers used in RT-qPCR; Table S2: DEGs associated with ABC transport; Table S3: DEGs associated with Entner–Doudoroff pathway and Phosphotransferase system; Table S4: DEGs associated with TCA cycle; Table S5: DEGs associated with Glycerol metabolism; Table S6: DEGs associated with Amino acid and Fatty acid metabolism; Table S7: DEGs associated with Oxidative phosphorylation; Table S8: DEGs associated with DNA replication and DNA repair; Table S9: DEGs associated with Quorum Sensing System; Table S10: DEGs associated with Chemotaxis system and Flagellar movement; Table S11: DEGs associated with Two-Component system.
