# Supplementary material for: Enhancing Benzo[a]pyrene Degradation by Pantoea dispersa MSC14 through Biostimulation with Sodium Gluconate: Insights into Mechanisms and Molecular Regulation
Source: Microorganisms. 2024 Mar 15;12(3):592. doi: 10.3390/microorganisms12030592 (PMC10975679; doi:10.3390/microorganisms12030592)
Supplement: Supplementary file 1 [file microorganisms-12-00592-s001.zip › Table S1.pdf]

Table S1 Primers used in RT-qPCR

Gene ID	Gene name	Forward primer sequence (5′-3′)	Reverse primer sequence (5′-3′)
K7H94_RS09205	PIR	CGGTCCCTTTACCATTCGC	TGCCGTTGAGGGCGAGAG
K7H94_RS19350	adh	TAGCCTATGGCGGATTGTT	CCGGTGCGATGACTTTATG
K7H94_RS13370	pht5	TGTCGTTCAGCCGCTATGC	GGTAACGCCGATTCTTTTG
K7H94_RS13920	tesC	AGATGCCGATGCCTTTCACT	CCACCAGCGTGTTGTCTCC
K7H94_RS02335	yfiH	GCTATGAGCTGGGCGTCG	ATTGCCTGCGGGTTGTGC
K7H94_RS11360	flgB	AACATTGCCAACGCCGATAC	TCCAGTGCGGGCTGTGAGT
K7H94_RS06390	gyrA	CCAACATTCCGCCTCACAAC	TAAATCCGGCCCGGTAATG
