# Supplementary figures and images for: Enhancing Benzo[a]pyrene Degradation by Pantoea dispersa MSC14 through Biostimulation with Sodium Gluconate: Insights into Mechanisms and Molecular Regulation
Source: Microorganisms. 2024 Mar 15;12(3):592. doi: 10.3390/microorganisms12030592 (PMC10975679; doi:10.3390/microorganisms12030592)

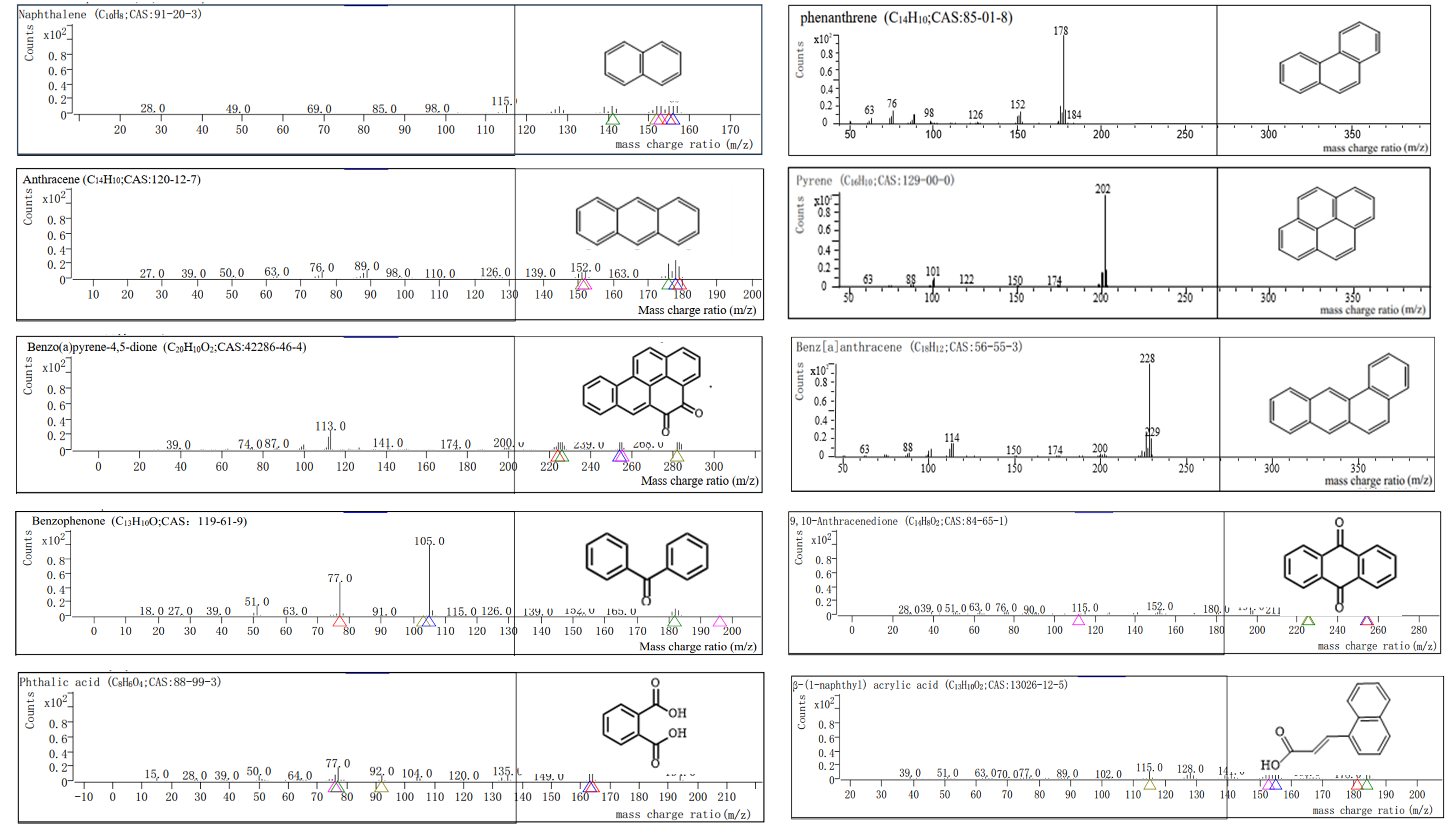

Supplement: Supplementary file 1 [file microorganisms-12-00592-s001.zip › Figure S1.tif]

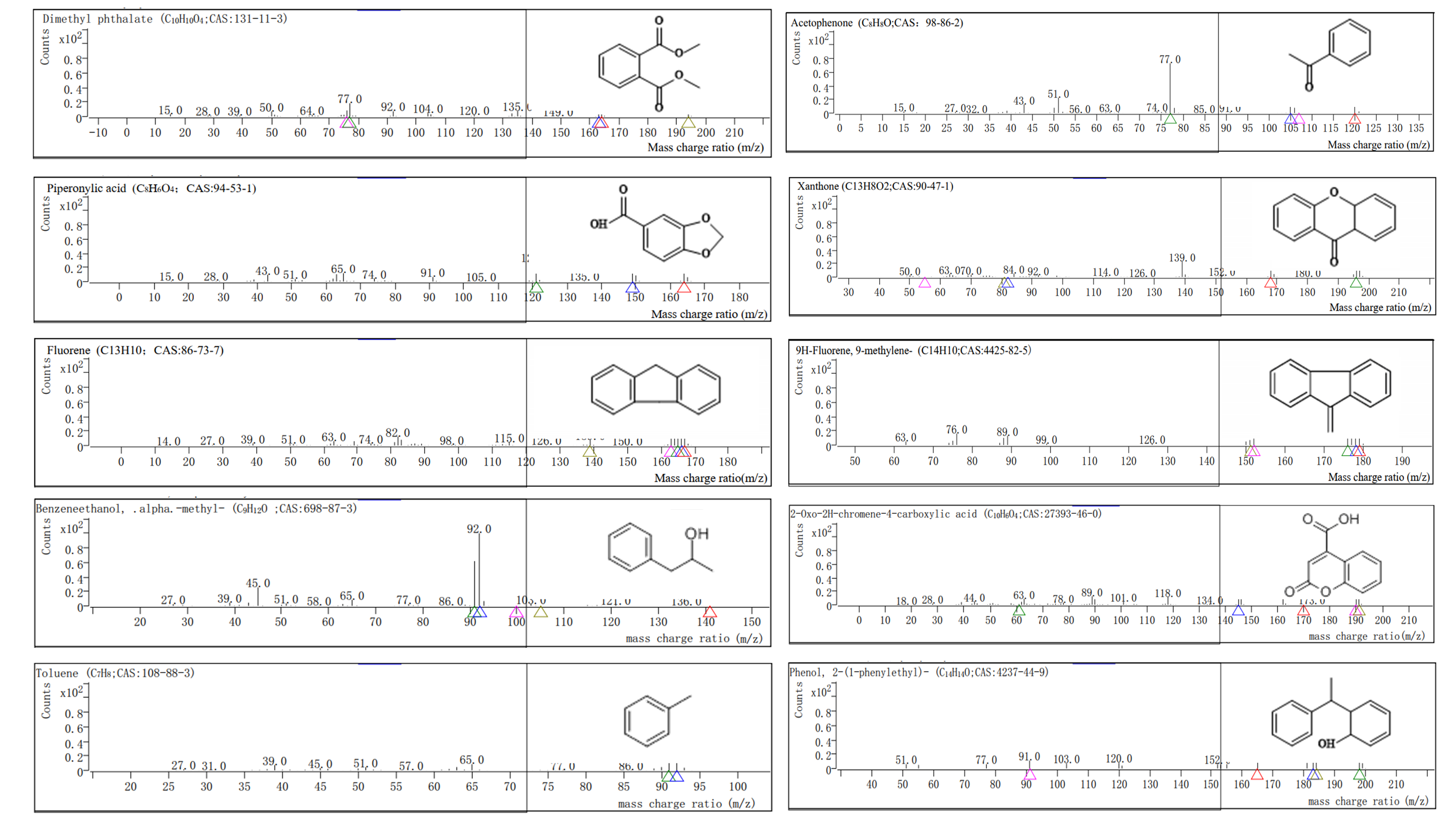

Supplement: Supplementary file 1 [file microorganisms-12-00592-s001.zip › Figure S2.tif]
